# Factor Structure of the Internet Addiction Test in Online Gamers and Poker Players

**DOI:** 10.2196/mental.3805

**Published:** 2015-04-22

**Authors:** Yasser Khazaal, Sophia Achab, Joel Billieux, Gabriel Thorens, Daniele Zullino, Magali Dufour, Stéphane Rothen

**Affiliations:** ^1^ Geneva University Hospitals Geneva Switzerland; ^2^ Laboratory for Experimental Psychopathology Psychological Sciences Research Institute Catholic University of Louvain Louvain-la-Neuve Belgium; ^3^ Sherbrooke University Montreal, QC Canada

**Keywords:** Internet addiction, Internet Addiction Test (IAT), poker players, World of Warcraft, massively multiplayer online role playing, validation, factorial structure

## Abstract

**Background:**

The Internet Addiction Test (IAT) is the most widely used questionnaire to screen for problematic Internet use. Nevertheless, its factorial structure is still debated, which complicates comparisons among existing studies. Most previous studies were performed with students or community samples despite the probability of there being more problematic Internet use among users of specific applications, such as online gaming or gambling.

**Objective:**

To assess the factorial structure of a modified version of the IAT that addresses specific applications, such as video games and online poker.

**Methods:**

Two adult samples—one sample of Internet gamers (n=920) and one sample of online poker players (n=214)—were recruited and completed an online version of the modified IAT. Both samples were split into two subsamples. Two principal component analyses (PCAs) followed by two confirmatory factor analyses (CFAs) were run separately.

**Results:**

The results of principal component analysis indicated that a one-factor model fit the data well across both samples. In consideration of the weakness of some IAT items, a 17-item modified version of the IAT was proposed.

**Conclusions:**

This study assessed, for the first time, the factorial structure of a modified version of an Internet-administered IAT on a sample of Internet gamers and a sample of online poker players. The scale seems appropriate for the assessment of such online behaviors. Further studies on the modified 17-item IAT version are needed.

## Introduction

As the main medium of modern life, the Internet is used in a wide range of human activities. This expansion has numerous benefits, including its use for social, psychological, and medical purposes, as shown by a wide range of studies on eHealth [1-11]. In parallel, however, serious concerns have been raised related to problematic or excessive uncontrolled Internet use [12-15].

In recent years, several studies proposed incorporating Internet addiction as a new diagnosis into the Diagnostic and Statistical Manual of Mental Disorders (DSM) [16,17]. However, it was recently decided that empirical research is still too scarce to allow its inclusion as a new psychiatric disorder in the fifth edition (DSM-5). The validity of the Internet addiction construct is challenged by both theoretical and empirical concerns. For some researchers, Internet addiction is an umbrella construct that encompasses a variety of different dysfunctional behaviors related to involvement in different online activities that do not necessarily coexist (eg, video games, cybersex, social networks, online gambling). Internet addiction thus seems to pertain to specific domains of use (ie, gaming, cybersex, social networks, or gambling) rather than to a general pattern of Internet use [12,18]. From this perspective, Internet gaming disorder (IGD) was proposed as a new condition and included in Section III of the DSM-5. The goal of Section III is to foster research on the conditions included therein [19].

With this potential new diagnosis, an important challenge is to develop assessment tools that are able to capture the specificity of this phenomenon, not only in terms of presence or absence of a given diagnostic, but also in terms of gradient severity. Since the initial research on Internet addiction, several psychometric measures have been developed [20-22]. One of the most translated tool is the Internet Addiction Test (IAT) [23]. The IAT is considered to be one of the instruments providing the most relevant clinical information about Internet gaming addiction [22,24]. The IAT has been moderately to highly correlated with a number of other measures of excessive Internet use, such as the Compulsive Internet Use Scale [25-28], the Generalized Problematic Internet Use Scale [29], the Revised Chen Internet Addiction Scale [30], and the Korean Internet Addiction Scale [31].

Despite the large diffusion of the IAT for research purposes, there is wide disagreement related first, to its factor structure [29] and second, to possible problematic items. In regard to the factor structure (see Multimedia Appendix 1), one to six factors were reported across studies published in English [27-45]. Furthermore, when similar numbers of factors were found, differences were observed in the distribution of the items on the factors. In addition, when more than one factor was extracted, a number of studies reported high correlations between factors [30,41,43], or a low contribution of some factors to the percentage of explained variance [27,35,42-45].

The issues related to the factorial structure of the IAT were possibly complicated by specific item-related concerns [28]. This led some authors to modify or discard items [27,28,30,31,34,41,45]. The most repeatedly reported concerns were the following:

1. Item 4—“How often do you form new relationships with fellow online users?”—has problematic loadings in a number of studies [27,28,36]. The recent rise of social networking, which widely disseminated this phenomenon, was suggested as a plausible explanation.

2. Item 6—on consequences on school work—and Item 8—related to job performance—ask about similar fields. However, the answer may differ depending on the participant’s understanding and on the specific status of the participant. Unsurprisingly, covariance was repeatedly found between these items [29,30,32,33]. Some authors removed Item 8 [28,34,44,45] or Item 6 [45] after analysis.

3. A similar type of overlap was shown between Item 3—“How often do you prefer the excitement of the Internet to intimacy with your partner?”—and Item 19—“How often do you choose to spend more time online over going out with others?” This lead some authors to discard one of the items [28] or to modify Item 3 [27] because of the influence of lifestyle (ie, having a partner or not) on the answer options.

4. In contrast with the other IAT components, Item 7—“How often do you check your email before something else that you need to do?”—is not related to the Internet in general, but to a specific use (ie, emails). Concerns were reported in a number of studies [27-29,31,35,41,44], in part because of the wide dissemination of email, and in part because of changes in this type of communication—the automatic notification of its reception, thus, no need to “check”, and its progressive replacement by social media. The item was deleted by some authors [28,30,41] and modified by others [31].

5. As a result of permanent Internet access (ie, without a specific need to log in), a rewording of Item 14 was proposed, as follows: “How often do you lose sleep due to being online late at night?” [28].

As shown, some of these IAT items involved specific patterns of life, such as being employed or being in a relationship. The “not applicable” answer option was probably included for this reason. It was, however, considered to be problematic by some authors [28] and was not systematically used.

In addition to conflicting results on the structure and certain items of the IAT, the psychometric characteristics were mostly assessed with students or community samples (Multimedia Appendix 1).

To our knowledge, no previous studies have assessed the psychometric characteristics of the instrument specifically for users of a given Internet application such as Internet games or gambling sites, despite the wide use of the scale, with or without modification, in studies related to these specific patterns of use [28,46,47].

In the context of increasing interest in possible Internet addiction-related disorders—with common involvement of gaming and gambling—and the emergence of the DSM-5 concept of the Internet gaming disorder, the use of a modified IAT for assessment of online gaming and online gambling may be worthwhile. Textbox 1 shows a modified version of the IAT for Internet game use.

Moreover, the lack of published studies on the psychometrical properties of the IAT on samples of gamers or gamblers appears to be an important weakness. This is of particular importance considering the increasing resemblance between gambling and gaming [48] and the possible links between related online activities (ie, massively multiplayer online role-playing games, online poker) and patterns of problematic Internet use [10,49-53].

The main goal of this study, therefore, was to investigate the factorial structure of the French version of the IAT modified for Internet gaming—or gambling—when used online, using samples of Internet gamers and Internet gamblers.

Table 1 shows the correspondence of the IAT items in Textbox 1 with the DSM-5 gaming and gambling criteria.

**The Internet Addiction Test modified for Internet game use.**

IAT items modified for Internet game use:

1. How often do you find that you stay in-game longer than you intended?

2. How often do you neglect household chores to spend more time in-game?

3. How often do you prefer the excitement of the game to intimacy with your partner?

4. How often do you form new relationships with fellow game users?

5. How often do others in your life complain to you about the amount of time you spend in-game?

6. How often do your grades or school work suffer because of the amount of time you spend in-game?

7. How often do you check your email before something else that you need to do?

8. How often does your job performance or productivity suffer because of the game?

9. How often do you become defensive or secretive when anyone asks you what you do in-game?

10. How often do you block out disturbing thoughts about your life with soothing thoughts about the game?

11. How often do you find yourself anticipating when you will go in-game again?

12. How often do you fear that life without the game would be boring, empty, and joyless?

13. How often do you snap, yell, or act annoyed if someone bothers you while you are in-game?

14. How often do you lose sleep due to late-night log-ins?

15. How often do you feel preoccupied with the game when offline, or fantasize about being in-game?

16. How often do you find yourself saying "just a few more minutes" when in-game?

17. How often do you try to cut down the amount of time you spend in-game and fail?

18. How often do you try to hide how long you've been in-game?

19. How often do you choose to spend more time in-game over going out with others?

20. How often do you feel depressed, moody, or nervous when you are offline, which goes away once you are back in-game?

**Table 1:** Internet Addiction Test item correspondence with the Diagnostic and Statistical Manual of Mental Disorders, fifth edition, gaming and gambling disorders criteria.

Internet gaming disorder: proposed DSM-5 criteria	Gambling disorder: DSM-5 criteria
Preoccupation with Internet games (11, 15)^a^	Preoccupation with gambling (11, 15)
Withdrawal symptoms when Internet gaming is taken away (20)	Withdrawal (restless or irritable when attempt to cut down or stop gambling) (20)
Tolerance (the need to spend increasing amounts of time engaged in Internet games)^b^	Tolerance (needs to gamble with an increasing amount of money)^b^
Unsuccessful attempts to reduce or stop Internet game participation (1, 16, 17)	Unsuccessful attempts to reduce or stop gambling (1, 16, 17)
Loss of interest in other activities (3, 7, 19)	N/A^c^
Continues excessive use of Internet games despite problems (2, 6, 8, 14)	N/A
Deceives others regarding the amount of Internet gaming (5, 9, 13, 18)	Lies to conceal the importance of gambling involvement (9, 18)
Use of Internet games to escape from adverse moods (10)	Escape (often gambles when feeling distressed) (10)
Has jeopardized or lost relationships or opportunities due to excessive Internet gaming (8)	Has jeopardized or lost relationships or opportunities due to excessive gambling (8)
N/A	Relies on others to provide money to relieve the financial situations caused by gambling^b^
N/A	After losing money gambling, often returns to get even (“chasing” one’s losses)^b^
Number of criteria: 5 or more	Number of criteria: 4 or more
Time criteria: 12 months or more	Time criteria: 12 months or more
Exclusion criteria: Internet use not related to online games is not “analogous to Internet gaming disorder”	Exclusion criteria: The behavior is better explained by a manic episode

^a^The suggested IAT items from Textbox 1 for each criteria are shown in parentheses.

^b^Not associated with an IAT item.

^c^Not applicable (N/A).

## Methods

### Participants

Two samples were used in this study: a French-speaking sample of World of Warcraft (WoW) players and a French-speaking sample of Internet poker players. These two samples completed the same modified version of the IAT. The ethical committee of the Department of Psychology of the University of Geneva—for the WoW sample—and the ethical committee of the Geneva University Hospitals—for the poker players sample—approved the study.

### World of Warcraft Players Sample

The WoW sample was taken from a larger study on the relationships between players’ self-reported motives to play and their in-game behaviors [46]. To participate in this study, an individual had to be a French-speaking WoW player and at least 18 years old. Participants were recruited through advertisements posted in dedicated French-language forums: a guilds forum, an official Blizzard WoW forum, and more general online and video games forums. Some participants also joined the study after hearing about it through the local press or television interviews. All participants gave their online consent prior to starting the online survey. The sample included 920 subjects who completed the French-language translation of the IAT [32] modified for online gaming. The mean age of IAT completers was 26.0 years (SD 7.8) and 807 of the 920 subjects (87.7%) were men.

### Poker Players Sample

#### Overview

The poker players sample was taken from a study on online gambling. Inclusion criteria included playing online poker, speaking French, and being at least 18 years old. Participants were recruited through advertisements posted in dedicated French-language forums on online gambling or poker. All participants gave their online consent. The sample of poker players included 442 participants, of whom 214 (48.4%) completed the IAT. The mean age of IAT completers was 31.9 years (SD 9.5) and 425 of the 442 participants (96.2%) were men.

#### Measurements

All participants—WoW gamers and poker players—completed the same modified IAT. The scale is a 20-item auto-questionnaire [23] rated on a 5-point Likert scale from 1 (rarely) to 5 (always) with a maximum total score of 100. The rating also includes a “not applicable” option that has a rating of 0. In this study, we used the validated French version [32], which was adapted by replacing words directly related to the Internet with words describing the specific activity (eg, “How often do you find that you stay online longer than you intended?” was replaced with “How often do you find that you stay in-game longer than you intended?”).

### Data Analyses

Because no clear factor structure has emerged in the literature and because different studies that found the same number of factors were inconsistent regarding factor loadings, we decided to assess the factor structure underlying this questionnaire from scratch (ie, without imposing a specific model or number of factors). In order to achieve this goal, both samples were randomly split into two subsamples of half of the size of the original ones (ie, 107 subjects for the poker sample and 460 for the WoW sample). Two principal component analyses (PCAs) were first performed on the first subsamples separately. With the discrete nature of the IAT items, PCA is preferred over factor analysis since PCA does not assume any particular multivariate model, which is not the case for factor analysis [54]. Moreover, it is known that when the same number of factors or components are extracted, both techniques yield highly similar results [55]. The number of components to extract was determined by the scree test [56] and by Velicer’s minimum average partial (MAP) test done on the correlation matrix [57]. The reliability of the questionnaire was assessed by using the Cronbach alpha coefficient [58], which is a measure of internal consistency.

In a second step, two confirmatory factor analyses (CFAs) were conducted to validate the structure that emerged from the PCA. The CFAs were run on the second subsamples. For the same reasons that PCA was preferred, the unweighted least-square method was chosen as the procedure for estimation. Four preestablished criteria were selected as indicators of the goodness of fit to the data: (1) goodness-of-fit index >.90 [59], (2) adjusted goodness-of-fit index >.80 [59], (3) normed-fit index >.90 [60], and (4) root-mean-square error <.08. The use and cutoff of the goodness-of-fit index and the adjusted goodness-of-fit index were recommended by Cole [61], the normed-fit index by Bentler and Bonnet [60], and the root-mean-square error by Hu and Bentler [62].

The PCA was done with R 3.1.0, using *psych* and *bootstrap* packages, and the CFA was done with AMOS 21.0.0 [63].

## Results

### Principal Component Analysis

The MAP test and the scree test clearly suggested in both subsamples that one component be extracted. In order to evaluate the stability of the PCA, a bootstrap technique [64] was performed first with the MAP test, which confirmed the one-factor solution—among the 1000 bootstrap samples, 60.10% (601) and 84.60% (846) suggested retaining one factor in the poker and in the WoW subsamples, respectively. The bootstrap was also applied to factor loadings of the PCA. Items 4 and 6 had a very low loading on the factor, confirmed by the confidence intervals based on the bootstrap in both subsamples, suggesting that these questions may not be well-suited for the questionnaire (Table 2).

The percentage explained variance (95% CI) was 41.6 (31.6-51.1) for poker players and 36.1 (32.6-39.8) for WoW players. The reliability, as reported by Cronbach alpha (95% CI), was .92 (.88-.95) for poker players and .90 (.88-.92) for WoW players.

**Table 2:** The 20-item Internet Addiction Test results from principal component analysis.

Items	Estimated factor loadings (95% bootstrap CI)	
	Poker players (n=107)	WoW players (n=460)
1. How often do you find that you stay in-game longer than you intended?	.65 (.52-.75)	.49 (.38-.58)
2. How often do you neglect household chores to spend more time in-game?	.67 (.54-.77)	.73 (.68-.77)
3. How often do you prefer the excitement of the game to intimacy with your partner?	.71 (.54-.84)	.41 (.29-.52)
4. How often do you form new relationships with fellow game users?	0 (-.20 to .20)	.26 (.14-.37)
5. How often do others in your life complain to you about the amount of time you spend in-game?	.73 (.59-.86)	.61 (.53-.67)
6. How often do your grades or school work suffer because of the amount of time you spend in-game?	.29 (.04-.55)	.50 (.41-.59)
7. How often do you check your email before something else that you need to do?	.60 (.35-.76)	.67 (.60-.73)
8. How often does your job performance or productivity suffer because of the game?	.63 (.38-.80)	.66 (.57-.73)
9. How often do you become defensive or secretive when anyone asks you what you do in-game?	.65 (.42-.82)	.52 (.42-.61)
10. How often do you block out disturbing thoughts about your life with soothing thoughts about the game?	.72 (.57-.84)	.68 (.62-.74)
11. How often do you find yourself anticipating when you will go in-game again?	.57 (.39-.71)	.69 (.64-.73)
12. How often do you fear that life without the game would be boring, empty, and joyless?	.56 (.32-.73)	.64 (.54-.71)
13. How often do you snap, yell, or act annoyed if someone bothers you while you are in-game?	.62 (.38-.77)	.63 (.55-.70)
14. How often do you lose sleep due to late-night log-ins?	.68 (.48-.80)	.64 (.58-.69)
15. How often do you feel preoccupied with the game when offline, or fantasize about being in-game?	.64 (.46-.77)	.69 (.62-.74)
16. How often do you find yourself saying "just a few more minutes" when in-game?	.65 (.45-.78)	.57 (.50-.63)
17. How often do you try to cut down the amount of time you spend in-game and fail?	.77 (.62-.87)	.52 (.41-.61)
18. How often do you try to hide how long you've been in-game?	.80 (.64-.89)	.55 (.45-.63)
19. How often do you choose to spend more time in-game over going out with others?	.69 (.53-.81)	.64 (.56-.71)
20. How often do you feel depressed, moody, or nervous when you are offline, which goes away once you are back in-game?	.79 (.67-.86)	.72 (.64-.77)

### Reliability

Cronbach alpha was above .90 in both subsamples, which was found to be excellent. It is worth noting that when Item 4 or Item 6 were removed, Cronbach alpha increased from .92 to .93 for poker players and from .90 to .91 for WoW players.

### Confirmatory Factor Analysis

According to the cutoff defined above, all four goodness-of-fit indices were considered excellent in both subsamples (Table 3).

### Shorter Version of the Internet Addiction Test: 17-Item Questionnaire

Because some items had low loadings and some questions had more missing values than occurred in the rest of the questionnaire, we performed additional investigations. In particular, Question 4—“How often do you form new relationships with fellow game users?”—seemed somewhat outdated and thus no longer relevant. Moreover, it had a low loading and decreased Cronbach alpha. Therefore, we decided to remove it.

Since Question 6—“How often do your grades or school work suffer because of the amount of time you spend in-game?”—is more suitable for school-aged persons, whereas Question 8—“How often does your job performance or productivity suffer because of the game?”—is more adapted to adults, we decided to merge the two questions into one. This new question addresses the consequences for the participant’s principal occupation, either school or work, preventing the participant from omitting the answer because it is not applicable. For the same reasons, we also merged Question 3—“How often do you prefer the excitement of the game to intimacy with your partner?”—and Question 19—“How often do you choose to spend more time in-game over going out with others?”

These modifications led to a 17-item questionnaire. Despite the fact that this version had not been tested on new subjects, we performed the same analyses as we did for the original questionnaire—randomly split both samples into two subsamples, running MAP, PCA, and CFA—by using the data at hand. For the merged questions, we decided to create two new items as follows: use the maximum mark of the IAT Item 3 and Item 19, as well as of Item 6 and Item 8, for each participant when both questions have been answered, or use the mark of only a single answered question.

In accordance with these modifications, the WoW sample size of IAT completers increased from 920 to 942 subjects, and the French-speaking poker sample size increased from 214 to 232. As expected, the results with this new method of coding the questionnaire led to the same conclusions regarding the number of components to extract—the bootstrapped MAP test suggested retaining only one factor in 74.2% and 95.6% of the poker and the WoW subsamples, respectively—regarding the factorial solution with the benefit of avoiding low loadings (Table 4), and regarding Cronbach alpha. Moreover, every fit index from the CFA was the same or slightly better (Table 3).

The percentage explained variance (95% CI) was 48.4 (37.5-58.1) for poker players and 37.3 (33.4-41.0) for WoW players. The reliability, as reported by Cronbach alpha (95% CI), was .93 (.90-.96) for poker players and .89 (.87-.91) for WoW players.

**Table 3:** Results from unweighted least-square confirmatory factor analysis.

Fit indices	Unweighted least squares			
	Poker players		WoW players	
	IAT20^a^ (n=107)	IAT17^b^ (n=116)	IAT20 (n=460)	IAT17 (n=471)
Root-mean-square residual	.08	.07	.08	.08
Goodness-of-fit index	.97	.97	.97	.97
Adjusted goodness-of-fit index	.97	.97	.96	.97
Normed-fit index	.96	.96	.95	.96

^a^The 20-item Internet Addiction Test (IAT20).

^b^The 17-item Internet Addiction Test (IAT17).

**Table 4:** The 17-item Internet Addiction Test results from principal component analysis.

Items	Estimated factor loadings (95% bootstrap CI)	
	Poker players (n=107)	WoW players (n=460)
1. How often do you find that you stay in-game longer than you intended?	.63 (.49-.73)	.51 (.41-.60)
2. How often do you neglect household chores to spend more time in-game?	.75 (.64-.84)	.70 (.65-.75)
Item 3 plus Item 19	.78 (.68-.86)	.64 (.57-.70)
5. How often do others in your life complain to you about the amount of time you spend in-game?	.68 (.51-.79)	.54 (.47-.62)
Item 6 plus Item 8	.65 (.53-.75)	.64 (.56-.71)
7. How often do you check your email before something else that you need to do?	.67 (.47-.79)	.69 (.62-.75)
9. How often do you become defensive or secretive when anyone asks you what you do in-game?	.70 (.48-.82)	.46 (.35-.55)
10. How often do you block out disturbing thoughts about your life with soothing thoughts about the game?	.76 (.60-.87)	.67 (.60-.73)
11. How often do you find yourself anticipating when you will go in-game again?	.59 (.42-.72)	.68 (.63-.73)
12. How often do you fear that life without the game would be boring, empty, and joyless?	.67 (.49-.80)	.59 (.49-.68)
13. How often do you snap, yell, or act annoyed if someone bothers you while you are in-game?	.61 (.41-.76)	.57 (.48-.65)
14. How often do you lose sleep due to late-night log-ins?	.63 (.43-.77)	.66 (.59-.71)
15. How often do you feel preoccupied with the game when offline, or fantasize about being in-game?	.69 (.52-.80)	.67 (.61-.73)
16. How often do you find yourself saying "just a few more minutes" when in-game?	.64 (.46-.76)	.55 (.47-.62)
17. How often do you try to cut down the amount of time you spend in-game and fail?	.73 (.55-.85)	.50 (.39-.60)
18. How often do you try to hide how long you've been in-game?	.79 (.63-.89)	.54 (.45-.62)
20. How often do you feel depressed, moody, or nervous when you are offline, which goes away once you are back in-game?	.81 (.63-.88)	.69 (.63-.75)

## Discussion

### Principal Findings

This study is the first to assess, to our knowledge, the psychometric characteristics, and specifically the factorial structure, of the IAT in online samples of WoW gamers and poker players. The main finding is that the one-factor model of the IAT has good psychometric properties and fits the data well in these samples.

In consideration to both the important discrepancies in the factorial solutions found in the previous studies on the IAT and the inconsistencies in the items included in a given factor in studies with a similar number of factors (Multimedia Appendix 1), the study at hand used an exploratory approach (ie, PCA) rather than an assessment of multiple competitive CFA models. 

Although heterogeneous results were found regarding the one-factor solution in previously reported studies, it was considered the best factor solution, or as possibly an acceptable factor solution (Multimedia Appendix 1) [32-34,37-39,44,45].

As suggested elsewhere [39], the instability of the IAT factorial structure is possibly linked to its multiple strongly correlated facets. The variability of the samples studied, as well as some specific item-related aspects, may have contributed to the phenomenon. This consideration led some authors to propose modifications of the IAT [27,28,30,31,34,39].

The modified 17-item IAT scale proposed here (Table 4) also has a one-factor structure. The modification and combination of items related to a specific consequence on lifestyle (eg, students, workers) may reduce the sample variability of the proposed tool. The changes related to Item 6—impact on school—and Item 8—impact on occupation—are concordant with other findings [29,30,32,33], which previously led some authors to remove one of the two items [28,34,44,45]. Removing Item 6 or 8, however, may cause variable results, depending on the population studied (ie, students or workers). The modification proposed here may allow the answer to the combined item to depend less on the population studied. Similarly, observations about Item 3 and Item 19 of the IAT led some authors to discard or modify one of these items [28]. Again, the 17-item IAT may reduce sample-related changes in the scale characteristics.

The problematic loading of Item 4 was also reported in some studies [27,28,31,36]. In consideration of the emergence and wide dissemination of online social networks, this item appears to be less pertinent for the assessment of Internet addiction. In contrast with the results of previous studies, however, those of this study did not indicate specific problems related to Item 7—“How often do you check your email before something else that you need to do?” [27,28,35,41,44]—or Item 14—“How often do you lose sleep due to being online late at night?” [28]. Because of the concerns related to Item 7, some authors chose to delete it [28,30,41], whereas others proposed replacing “emails” with “Internet” [31]. In relation to this, the modification proposed by Lee and colleagues [31] seems relevant for further studies. The word “Internet” could be replaced with the specific Internet usage studied (ie, game). An interesting rewording of Item 14 was also proposed elsewhere [28] as follows: “How often do you lose sleep due to being online late at night?” The development of the Internet has led to possible permanent access without a requirement to log in. The lack of a specific problem with Items 7 and 14 in this study was possibly due to the age of the sample participants, for whom checking email or logging in were more familiar concepts than they were to younger people. The 17-item IAT could be adapted to include the modification proposed by Lee et al [31] for Item 7 and to that proposed by Pawlikowski et al [28] for Item 14 (Table 4).

The 17-item IAT scale (Textbox 2) and the 20-item IAT (Textbox 1, Table 1) offer interesting coverage of the main items of the DSM-5 criteria for IGD, with the exception of tolerance and time frame, which were not covered by these tools. As shown in Textbox 2, it is easy to add a time frame using a question such as “During the last year, how often…?”

As there is an absence of questions related to tolerance in the original IAT, it is then also the case for the proposed data-driven 17-item IAT. A further study may add questions specifically related to tolerance in order to assess the full range of symptoms proposed by the DSM-5 criteria for IGD.

The relatively good coverage of the 17-item IAT of the DSM-5 criteria for IGD is a possible advantage in comparison to other shorter forms of the IAT that lead, for example, to withdrawal of the escape-related item. Unsurprisingly, the 17-item IAT scale and the original IAT do not cover items related to the financial conflict-related items of the DSM-5 criteria for gambling disorder.

One of the strengths of this paper is related to the design of a 17-item IAT scale through a data-driven approach, rather than a priori choices.

Despite the possible lack of items linked to specific Internet use, it appears from this study that the original IAT and the 17-item IAT are interesting assessment tools for disorders related to excessive Internet use. In particular, the results assessed in this study add to the validity of IAT use with specific rewording, such as replacing “Internet” with “game.”

**Proposed new 17-item Internet Addiction Test gaming questionnaire. ^a^: Possible modification to: “How often do you stay in-game before something else that you need to do?“ ^b^: Possible modification to: “How often do you lose sleep due to being in-game late at night?“**

IAT items modified for the 17-item IAT (a time frame should be added to the top of the scale, such as “During the last year, how often….?”):

1. How often do you find that you stay in-game longer than you intended?

2. How often do you neglect household chores to spend more time in-game?

3. How often do you prefer the excitement of the game to intimacy with your partner, or to spend more time in-game over going out with others?

4. How often do others in your life complain to you about the amount of time you spend in-game?

5. How often do your grades or your school work, or your job performance or productivity suffer because of the amount of time you spend in-game?

6. How often do you check your email^a^ before something else that you need to do?

7. How often do you become defensive or secretive when anyone asks you what you do in-game?

8. How often do you block out disturbing thoughts about your life with soothing thoughts about the game?

9. How often do you find yourself anticipating when you will go in-game again?

10. How often do you fear that life without the game would be boring, empty, and joyless?

11. How often do you snap, yell, or act annoyed if someone bothers you while you are in-game?

12. How often do you lose sleep due to late-night log-ins?^b^

13. How often do you feel preoccupied with the game when offline, or fantasize about being in-game?

14. How often do you find yourself saying "just a few more minutes" when in-game?

15. How often do you try to cut down the amount of time you spend in-game and fail?

16. How often do you try to hide how long you've been in-game?

17. How often do you feel depressed, moody, or nervous when you are offline, which goes away once you are back in-game?

### Limitations

The main limitations of this study include the representativeness of self-selected samples [65], the lack of direct assessment with the modified 17-item IAT version, and the lack of concomitant diagnostic interviews.

### Conclusions

Further studies may test the 17-item IAT among various samples in parallel with other psychopathological and Internet-related behavior assessments, including the use of the DSM-5 proposed criteria and other assessment tools, with possibly different or complementary coverage of the concept of Internet addiction [20,66-70]. The use of the original IAT, and possibly the 17-item IAT, may stimulate further research on IGD and Internet addiction. Due to the wide variety of Internet activities, such as Internet gambling and Internet gaming, it should be necessary to specify the assessed behavior (eg, WoW, other game, gambling) for each use of the 17-item IAT. Research in these fields seems to be of particular interest, as suggested by the recent inclusion of IGD in the DSM-5, Section III. The relevance of the proposed criteria in the DSM-5 needs to be confirmed, however, as some criteria, such as deception and tolerance, continue to be debated [17].

## Appendix

Multimedia Appendix 1 Summary of previous studies on the Internet Addiction Test factorial structure.

## Abbreviations

CFA: confirmatory factor analysis
DSM: Diagnostic and Statistical Manual of Mental Disorders
DSM-5: Diagnostic and Statistical Manual of Mental Disorders, fifth edition
IAT: Internet Addiction Test
IAT17: 17-item Internet Addiction Test
IAT20: 20-item Internet Addiction Test
IGD: Internet gaming disorder
MAP: minimum average partial
N/A: not applicable
PCA: principal component analysis
WoW: World of Warcraft
