# Supplementary material for: Factor Structure of the Internet Addiction Test in Online Gamers and Poker Players
Source: JMIR Ment Health. 2015 Apr 22;2(2):e12. doi: 10.2196/mental.3805 (PMC4607378; doi:10.2196/mental.3805)
Supplement: Multimedia Appendix 1 [file mental_v2i2e12_app1.pdf]

	Khazaal et al. 2008	Hawi 2013	Panayides et al. 2012	Payanides et al. 2012	Panayides et al. 2012
IAT version	Complete French (paper administration)	Complete Arabic (on-line administration)	Complete Greek version (paper administration)	19-item Greek version (issued from further analyses on the 20-item scale)	20-item modified Greek version (paper administration)
Sample	N: 246 adult university students or community volunteers	N: 871 intermediate and secondary school students	N: 604 high school students in Cyprus	N: 604 high school students (the 19 items are issued from analyses on the complete scale)	N: 604 high school students in Cyprus
1. How often do you find that you stay on-line longer than you intended?	1	1	1	1	1
2. How often do you neglect household chores to spend more time online?	1	1	1	1	1
3. How often do you prefer the excitement of the Internet to intimacy with your partner?	1	1	1	1	1
4. How often do you form new relationships with fellow online users?	1	1	1	1	1
5. How often do others in your life complain to you about the amount of time you spend online?	1	1	1	1	1
6. How often do your grades or school work suffer because of the amount of time you spend online?	1	1	1	1	1
7. How often do you check your e-mail before something else that you need to do?	1	1	1	1	1
8. How often does your job performance or productivity suffer because of the Internet?	1	1	1	X [a1]	X[a2]
9. How often do you become defensive or secretive when anyone asks you what you do online?	1	1	1	1	1
10. How often do you block out disturbing thoughts about your life with soothing thoughts of the Internet?	1	1	1	1	1
11. How often do you find yourself anticipating when you will go online again?	1	1	1	1	1
12. How often do you fear that life without the Internet would be boring, empty, and joyless?	1	1	1	1	1
13. How often do you snap, yell, or act annoyed if someone bothers you while you are online?	1	1	1	1	1
14. How often do you lose sleep due to late-night log-ins?	1	1	1	1	1
15. How often do you feel preoccupied with the Internet when off-line, or fantasize about being in-game?	1	1	1	1	1
16. How often do you find yourself saying "just a few more minutes" when online?	1	1	1	1	1
17. How often do you try to cut down the amount of time you spend online and fail?	1	1	1	1	1
18. How often do you try to hide how long you've been online?	1	1	1	1	1
19. How often do you choose to spend more time online over going out with others?	1	1	1	1	1
20. How often do you feel depressed, moody, or nervous when you are off-line, which goes away once you are back online?	1	1	1	1	1

[a1] Discarded item

[a2] Modified item as follows: "To what extent do you think you are addicted to the Internet?"

	Pontes et al. 2014	Tsimtsiou et al. 2013	Lai et al. 2013	Teo et al. 2014	Chang et al. 2008
IAT version	Complete Portuguese (paper and on-line administartion)	Complete Greek (paper administration)	Modified[b] Chinese	Modified[d] English version of IAT for adolescents	Complete Chinese and English versions shown together
Sample	N: 593 high school and university students (including on-line condition N: 53)	N: 151 postgraduate and undergraduate medical students	N: 844 Chinese adolescents (Grades 7 to 13)	N: 325 primary and secondary school students (Singapore)	N: 410 university undergraduates
1. How often do you find that you stay on-line longer than you intended?	1	2	2	1/F3	2
2. How often do you neglect household chores to spend more time online?	1	2	2	1/F2	2
3. How often do you prefer the excitement of the Internet to intimacy with your partner?	1	1	1	1/F1	1
4. How often do you form new relationships with fellow online users?	1	1	1	1/F2	1
5. How often do others in your life complain to you about the amount of time you spend online?	1	2	1	1/F1	1
6. How often do your grades or school work suffer because of the amount of time you spend online?	1	3	2	1/F2	2
7. How often do you check your e-mail before something else that you need to do?	1	3	X[c]	1/F2	X[e]
8. How often does your job performance or productivity suffer because of the Internet?	1	3	2	1/F2	2
9. How often do you become defensive or secretive when anyone asks you what you do online?	1	3	1	1/F1	1
10. How often do you block out disturbing thoughts about your life with soothing thoughts of the Internet?	1	1	3	1/F1	3
11. How often do you find yourself anticipating when you will go online again?	1	1	X[c]	1/F1	X[e]
12. How often do you fear that life without the Internet would be boring, empty, and joyless?	1	1	3	1/F1	3
13. How often do you snap, yell, or act annoyed if someone bothers you while you are online?	1	1	1	1/F1	1
14. How often do you lose sleep due to late-night log-ins?	1	2	3	1/F2	3
15. How often do you feel preoccupied with the Internet when off-line, or fantasize about being in-game?	1	1	1	1/F1	1
16. How often do you find yourself saying "just a few more minutes" when online?	1	2	2	1/F3	2
17. How often do you try to cut down the amount of time you spend online and fail?	1	2	2	1/F3	2
18. How often do you try to hide how long you've been online?	1	1	1	1/F1	1
19. How often do you choose to spend more time online over going out with others?	1	1	1	1/F1	1
20. How often do you feel depressed, moody, or nervous when you are off-line, which goes away once you are back online?	1	1	1	1/F1	1

[b] Modified for adolecents: "household chores" (Item 2) translated to "daily hassles"and "intimacy with partner" translated to "activities with companions"

[c] Discarded items

[d] Item 2 modified to "How often do you neglect homework to spend more time online?" Item 3 modified to "How often do you prefer the excitement of the Internet to time spent with your best friend?” Item 8 modified to "How often does your schoolwork suffer because of the Internet?"

[e] Discarded items after analysis

	Widyanto et al. 2011	Korkeila et al. 2010	Faraci et al. 2013	Ferraro et al. 2007	Jelenchick et al. 2012
IAT version	Complete English (online administration)	Complete Finnish (online administration)	Complete Italian	Complete Italian (online administration)	Complete English (online administration)
Sample	N: 225 Internet users	N: 1825 medical school or vocation college students	N: 485 college students	N: 236 Italian Internet chatters recruited online	N: 215 US college university students (and Facebook users) aged 18-20 years
1. How often do you find that you stay on-line longer than you intended?	2	1/F2	1/F2	5	2
2. How often do you neglect household chores to spend more time online?	2	1/F2	1/F2	2	2
3. How often do you prefer the excitement of the Internet to intimacy with your partner?	1	1/F1	1/F1	6	1
4. How often do you form new relationships with fellow online users?	2	1/F1	1/F1	1	1
5. How often do others in your life complain to you about the amount of time you spend online?	1	1/F1	1/F2	1	1
6. How often do your grades or school work suffer because of the amount of time you spend online?	2	1/F1	1/F2	4	2
7. How often do you check your e-mail before something else that you need to do?	2	1/F2	1/F2	3	2
8. How often does your job performance or productivity suffer because of the Internet?	1	1/F1	1/x	4	2
9. How often do you become defensive or secretive when anyone asks you what you do online?	1	1/F1	1/F2	1	1
10. How often do you block out disturbing thoughts about your life with soothing thoughts of the Internet?	1	1/F1	1/F1	6	1
11. How often do you find yourself anticipating when you will go online again?	1	1/F1	1/F1	3	1
12. How often do you fear that life without the Internet would be boring, empty, and joyless?	3	1/F1	1/F1	2	1
13. How often do you snap, yell, or act annoyed if someone bothers you while you are online?	3	1/F1	1/F1	1	1
14. How often do you lose sleep due to late-night log-ins?	3	1/F1	1/F1	2	2
15. How often do you feel preoccupied with the Internet when off-line, or fantasize about being in-game?	3	1/F1	1/F1	3	1
16. How often do you find yourself saying "just a few more minutes" when online?	2	1/F2	1/F2	1	2
17. How often do you try to cut down the amount of time you spend online and fail?	1	1/F2	1/x	5	2
18. How often do you try to hide how long you've been online?	1	1/F1	1/F1	1	1
19. How often do you choose to spend more time online over going out with others?	1	1/F1	1/F1	2	1
20. How often do you feel depressed, moody, or nervous when you are off-line, which goes away once you are back online?	3	1/ F1	1/F1	2	1

	Barke et al. 2012	Pawlikowski et al. 2013	Pawlikowski et al. 2013	Watters et al. 2013	Lee et al. 2013
IAT version	Complete German (online administration + paper administration)	Complete modified[f] German	Modified[g] short (12-item) German IAT	Complete English (paper administrarion)	Complete modified[h] Korean (paper administration)
Sample	N: 1041 online recruited sample (ON); 841 student off-line sample (OF)	N: 584 university students and non-students (recruitment through local advertisements at the university and Internet advertisements)	N: 465 university students and non-students (recruitment through local advertisements at the university and Internet advertisements)	N: 1948 Canadian high school students (16-18 years old)	N: 279 undergraduate students
1. How often do you find that you stay on-line longer than you intended?	2	1	1	1/ GF1	1
2. How often do you neglect household chores to spend more time online?	2	1	1	1/GF1	1
3. How often do you prefer the excitement of the Internet to intimacy with your partner?	1	x	x	1/GF2	2
4. How often do you form new relationships with fellow online users?	1	x	x	1/GF2	4
5. How often do others in your life complain to you about the amount of time you spend online?	1	x	x	1/x	1
6. How often do your grades or school work suffer because of the amount of time you spend online?	2	1	1	1/x	1
7. How often do you check your e-mail before something else that you need to do?	2	x	x	1/GF1	1
8. How often does your job performance or productivity suffer because of the Internet?	2	x	x	1/x	1
9. How often do you become defensive or secretive when anyone asks you what you do online?	1	2	2	1/GF2	4
10. How often do you block out disturbing thoughts about your life with soothing thoughts of the Internet?	1	x	x	1/GF2	4
11. How often do you find yourself anticipating when you will go online again?	1	x	x	1/GF2	2
12. How often do you fear that life without the Internet would be boring, empty, and joyless?	1	x	x	1/GF2	2
13. How often do you snap, yell, or act annoyed if someone bothers you while you are online?	1	2	2	1/GF2	3
14. How often do you lose sleep due to late-night log-ins?	2	1	1	1/x	1
15. How often do you feel preoccupied with the Internet when off-line, or fantasize about being in-game?	1	2	2	1/GF2	2
16. How often do you find yourself saying "just a few more minutes" when online?	2	1	1	1/GF1	1
17. How often do you try to cut down the amount of time you spend online and fail?	2	1	1	1/GF1	1
18. How often do you try to hide how long you've been online?	1	2	2	1/GF2	3
19. How often do you choose to spend more time online over going out with others?	1	2	2	1/GF2	2
20. How often do you feel depressed, moody, or nervous when you are off-line, which goes away once you are back online?	1	2	2	1/GF2	3

[f] After a first exploratory factor analysis, leading to 2 factors, the following items/low factor loadings (<.50) and /or high parallel loadings (>.20) were excluded: 4, 5, 7, 10, 12. Items 8 and 3 were excluded because of content overlap with other items with better loadings.
Item 3 was modified to the following ones: "How often do you prefer the excitement of the Internet to intimacy with your partner/friends?"

[g] Item 14 was modified as follows: "How often do you lose sleep due to being online late at night?"
Confirmatory factor analysis on the short modified version issued from the first analyses on the complete version.

[h] Item 7: The word “email” was modified to the more general "the Internet." Item 4, due to low correlation and overall internal consistency, exceeded that of total items when the item was deleted (exclude the item for the factor analysis).

	Chong Guan et al. 2012	Widyanto et al. 2004
IAT version	Complete modified[i] Malay version (paper administration)	Complete English (online administation)
Sample	N: 162 medical students	N: 86 adults recruited through the Internet
1. How often do you find that you stay on-line longer than you intended?	1	2
2. How often do you neglect household chores to spend more time online?	1	2
3. How often do you prefer the excitement of the Internet to intimacy with your partner?	2	6
4. How often do you form new relationships with fellow online users?	3	6
5. How often do others in your life complain to you about the amount of time you spend online?	4	5
6. How often do your grades or school work suffer because of the amount of time you spend online?	2	3
7. How often do you check your e-mail before something else that you need to do?	5	4
8. How often does your job performance or productivity suffer because of the Internet?	2	3
9. How often do you become defensive or secretive when anyone asks you what you do online?	2	3
10. How often do you block out disturbing thoughts about your life with soothing thoughts of the Internet?	1	1
11. How often do you find yourself anticipating when you will go online again?	1	4
12. How often do you fear that life without the Internet would be boring, empty, and joyless?	1	1
13. How often do you snap, yell, or act annoyed if someone bothers you while you are online?	4	1
14. How often do you lose sleep due to late-night log-ins?	1	2
15. How often do you feel preoccupied with the Internet when off-line, or fantasize about being in-game?	3	1
16. How often do you find yourself saying "just a few more minutes" when online?	1	5
17. How often do you try to cut down the amount of time you spend online and fail?	1	5
18. How often do you try to hide how long you've been online?	2	2
19. How often do you choose to spend more time online over going out with others?	2	1
20. How often do you feel depressed, moody, or nervous when you are off-line, which goes away once you are back online?	2	2

[i] How often do you neglect coursework/assignments (Il ne manque pas quelque chose ?) to spend more time online? Item 3 was modified as follows: "How often do you prefer the excitement of the Internet to intimacy with your partner/friends?" Item 10: “How often do you block disturbing thoughts about your life with soothing thoughts of the Internet?” The item was revised and translated to “How often do you ignore disturbing thoughts about your life withsoothing thoughts about the Internet?”
